# Geospatial characteristics of measles transmission in China during 2005−2014

**DOI:** 10.1371/journal.pcbi.1005474

**Published:** 2017-04-04

**Authors:** Wan Yang, Liang Wen, Shen-Long Li, Kai Chen, Wen-Yi Zhang, Jeffrey Shaman

**Affiliations:** 1 Department of Environmental Health Sciences, Mailman School of Public Health, Columbia University, New York, New York, United States; 2 Institute of Disease Control and Prevention, Academy of Military Medical Sciences, Beijing, P.R. China; 3 State Key Laboratory of Pollution Control and Resource Reuse, School of the Environment, Nanjing University, Nanjing, P.R. China; The Pennsylvania State University, UNITED STATES

## Abstract

Measles is a highly contagious and severe disease. Despite mass vaccination, it remains a leading cause of death in children in developing regions, killing 114,900 globally in 2014. In 2006, China committed to eliminating measles by 2012; to this end, the country enhanced its mandatory vaccination programs and achieved vaccination rates reported above 95% by 2008. However, in spite of these efforts, during the last 3 years (2013–2015) China documented 27,695, 52,656, and 42,874 confirmed measles cases. How measles manages to spread in China—the world’s largest population—in the mass vaccination era remains poorly understood. To address this conundrum and provide insights for future public health efforts, we analyze the geospatial pattern of measles transmission across China during 2005–2014. We map measles incidence and incidence rates for each of the 344 cities in mainland China, identify the key socioeconomic and demographic features associated with high disease burden, and identify transmission clusters based on the synchrony of outbreak cycles. Using hierarchical cluster analysis, we identify 21 epidemic clusters, of which 12 were cross-regional. The cross-regional clusters included more underdeveloped cities with large numbers of emigrants than would be expected by chance (*p* = 0.011; bootstrap sampling), indicating that cities in these clusters were likely linked by internal worker migration in response to uneven economic development. In contrast, cities in regional clusters were more likely to have high rates of minorities and high natural growth rates than would be expected by chance (*p* = 0.074; bootstrap sampling). Our findings suggest that multiple highly connected foci of measles transmission coexist in China and that migrant workers likely facilitate the transmission of measles across regions. This complex connection renders eradication of measles challenging in China despite its high overall vaccination coverage. Future immunization programs should therefore target these transmission foci simultaneously.

## Author summary

Measles is a highly infectious disease. Eradication of measles can nevertheless be achieved with vaccination of 90–95% of a population, as shown in theory and practice. In China, however, measles continues to infect thousands of people each year despite vaccination coverage above 95%. This conundrum challenges measles elimination in China and worldwide. Here we characterize the geospatial distribution of measles and epidemic connections among cities across China. Using incidence data reported during 2005–2014 for all 344 cities in China, we show that the municipal burden of measles differed substantially and some cities were highly connected and experienced synchronous outbreaks. We identify 14 cities that experienced endemic transmission during 2005–2010, and 21 transmission clusters, including 6 cross-regional clusters that link the less developed inland regions and the industrial east. We find that three transmission foci coexist in China—cities with large minority populations, inland cities with more emigrants, and mega industrial cities hosting more immigrants—and that migrant workers, connecting the latter two foci, likely facilitate measles transmission across regions. This complex connection, along with the differing disease burden among cities, renders measles elimination challenging in China despite the high overall vaccination rate. Future immunization programs should therefore target these three foci.

## Introduction

Measles is a highly contagious disease caused by the measles virus, a paramyxovirus, genus *Morbillivirus*. Before vaccine licensure in 1963, the disease infected virtually all children. Infection typically causes fever, runny nose, cough, red eyes, and sore throat, followed by a rash spreading over the body. While most cases recover, complications range from diarrhea, otitis media, pneumonia, encephalitis, seizures to death [1,2]. Since the implementation of mass vaccination programs, the number of measles infections has declined dramatically and many countries have declared elimination of the disease [2,3]. However, measles remains a leading cause of death among young children in developing regions, despite immense public health efforts [2,4]. In 2014, there were 114,900 measles deaths globally [5]. In addition, due to decreases in vaccination coverage and importation of cases through travel, the re-emergence of measles has become a concern in recent years for regions free of measles [2,3,6,7].

Prior to the introduction of measles vaccine in 1965, China recorded an average of 3–4 million measles cases per year, with incidence rates ranging from 200 to 1500 per 100,000 population [8]. The country mandated measles vaccination in 1978 and achieved vaccination rates above 95% by 2008 [8–10]. As a result, the average measles incidence rate dropped precipitously to 6.8 per 100,000 population during 2000–2009 [8]. In 2006, China committed to eliminate measles by 2012. To this end, China conducted synchronized nationwide supplementary immunization activities (SIA) in September 2010 in addition to routine vaccination programs, which vaccinated 97.5% of the targeted population [8,9]. However, the measles elimination goal was not achieved. While the annual number of confirmed cases reached record lows of 9,944 in 2011 and 6,276 in 2012, it resurged to 27,695 in 2013, 52,656 in 2014, and 42,874 in 2015 [11].

Theory predicts, and many observations have confirmed, that high vaccination coverage against measles (~90–95%) will lead to lower outbreak frequency, irregular outbreaks, and eventual elimination [12–18]. The continuous large outbreaks in China prompt speculation on the accuracy of reported vaccination coverage. To access the population profile of measles susceptibility, a recent study [19] tested 2213 people in Tianjin, a major Chinese municipality with regular measles outbreaks [20]; during 2011–2015, 87.8% tested positive for measles IgG antibody regardless of infection history and 91.8% were positive among those with a history of measles disease [5% (110) of the 2213 tested]. A multiplicative adjustment suggests an overall immunity of 95.6%, indeed above the 95% threshold. Such high measles immunity in the presence of the concurrent frequent outbreaks observed in China calls for improved understanding of measles transmission dynamics.

Previous studies have repeatedly identified three key factors that shape the transmission dynamics of measles [21–27]: 1) demographics, particularly birth rates and vaccination coverage, which determine rates of susceptibility, 2) seasonal factors that affect human density and contact patterns, e.g., epidemics during school-terms due to increased mixing among school-aged children in the pre-vaccination era, and 3) stochasticity inherent in dynamical systems, e.g. the community size should exceed a certain threshold (i.e. critical community size) to avoid extinction of transmission due to stochasticity. In this study, we focus on the geospatial characteristics of measles transmission and aim to identify the key population features that may be contributing to measles persistence in China. Using incidence and demographic data for all 344 cities in Mainland China, we analyze the spatiotemporal patterns of measles across China during the most recent decade and identify clusters and transmission paths of measles epidemics among the 344 cities. Our findings reveal key characteristics defining the cities with the most substantial disease burden. In addition, this study serves as a baseline analysis of measles dynamics in China prior to the implementation of the new nationwide two-child policy in 2016, which may lead to a baby boom and further complicate measles elimination in the years to come.

## Materials and methods

### Measles surveillance dataset

Measles surveillance data were compiled from the China Information System for Disease Control and Prevention (CISDCP)[28]. The CISDCP system is a web-based real time disease reporting system, established in 2004, that collects patient-case reports for all notifiable diseases, including measles, from all medical institutions in China. The system collects information on the age, gender, location of residence, and date of onset for each measles case. In the surveillance system, a suspected measles case was defined as any person with fever and rash and one or more symptoms of cough, coryza or conjunctivitis. For suspected measles cases, a serum specimen was collected and tested for measles-specific IgM or IgG, measles virus, or measles viral RNA. A confirmed case must meet one of the following criteria: 1) detection of measles-specific IgM in an acute serum specimen collected 3 days after rash onset, a ≥4 fold rise in measles-specific IgG based on testing of an acute serum specimen and a convalescent serum specimen, or a seroconversion between the two tests; 2) isolation of a measles virus or viral RNA; or 3) meet the clinical definition (fever of 38.3°C or higher, a maculopapular rash, and cough, coryza or conjunctivitis) and be epidemiologically linked to a confirmed case [9,29,30].

In this study, we restricted our analyses to confirmed cases. The dataset used included all confirmed cases reported from 1/1/2005 to 12/31/2014. While the total number of incident cases was large in China, measles incidence was sparse for the majority of counties. Therefore, we aggregated incidence records to the prefectural city level (i.e. 4-digit geo-division coding level) [31]; there were 344 cities in total during 2005–2014. As the generation time, i.e. the mean time interval between infection onset in a primary and secondary case, is approximately two weeks for measles [32], we aggregated the incidence records to bi-week intervals.

### Demographic, population mobility, and socioeconomic dataset

Data from the 2010 census in China [33] were used to produce the demographic profiles for each of the 344 cities. We utilized a number of demographic statistics, including 1) household registered population (similar to *local* population) and *total* population (i.e. local residents plus migrants); 2) percentages of urban, rural, or non-agricultural populations among the total population (same denominator for other percentages unless stated otherwise); 3) percentage of minority population (i.e. any of the 55 non-Han ethnic groups); 4) percentages of population aged 0–14, 15–64, or ≥65 yr; 5) birth rate, death rate, and natural growth rate (i.e. birth rate minus death rate); 6) average years of education and illiterate percentage of population aged ≥15 yr; and 7) population mobility data including percentages of migrants from the same county, other counties in the same province, or other provinces. These data were available at the county level and aggregated to the prefectural city level [31] to match the geospatial scale of measles incidence data. Because the population mobility data only recorded immigrants for each city but not emigrants, we used the difference between the *total* and *local* population size relative to the total population in each city as an indicator of net influx of migrants (i.e. immigrants minus emigrants, normalized by dividing the total population size). This estimate is reliable because all cities in China employ a strict household registration system (also known as ‘Hukou’), which records all individuals who are local residents (i.e. household registered population) and those from outside cities [34].

We also compiled data regarding municipal socioeconomic development from the China Statistical Yearbook for 2010 [35]. Data for gross domestic product (GDP) and per capita GDP were available for 340 of the 344 cities. Another indicator of socioeconomic development is the composition of GDP. The China Statistical Yearbook for 2010 included the shares of GDP from primary (i.e. agriculture and agriculture-related), secondary (i.e. manufacturing), and tertiary (i.e. service) industries for 289 of the 344 cities.

### Estimates of spatial temporal characteristics

To visualize the spatial-temporal pattern of measles transmission among the 344 cities, we scaled the biweekly incidence records for each city by dividing the city-specific maximum during 2005–2014. For each city, we computed the cumulative incidence (i.e. the total number of reported incident cases) and cumulative incidence rate (i.e. cumulative incidence per 100,000 population). Cumulative incidence and cumulative incidence rate were then mapped to each city to visualize the burden of measles infection across China.

Previous measles epidemiological studies [24,26,36] typically defined a “fadeout” event as zero measles cases reported for more than 2–4 weeks, and endemic transmission as ongoing transmission without any fadeout event. As the numbers of cases per bi-week in each city were in general low during our study period, we relaxed the definition of endemic transmission to allow one instance with no cases over 4 weeks (i.e. 2 consecutive bi-weeks). We identified cities with endemic measles during Jan 2005 –Aug 2010 (i.e. before the nationwide SIA) and Jan 2013 –Dec 2014 (i.e. after the resurgence of incidence), respectively.

### Hierarchical cluster analysis

We identified clusters of measles transmission using hierarchical cluster analysis (HCA) per two procedures. The first was a traditional HCA per the complete linkage method [37], which iteratively combines items being clustered based on similarities/dissimilarities between items. Here we used the absolute value of Pearson correlation coefficient (*r*) between the incidence time series as a measure of similarity. The complete linkage method combines groups of items based on the minimal similarity among all intergroup pairs, which results in more conservative clustering. This procedure was carried out using the “hclust” function in R (https://www.r-project.org), and therefore referred to as “hclust” hereafter.

As outbreaks occurring simultaneously in adjacent cities are likely parts of a single large outbreak, we developed an alternative HCA procedure to account for such regional clustering. In this procedure (referred to as “recursive merging” hereafter), we first computed the Pearson correlation coefficient of incidence time series for each of the city pair combinations (e.g. 344×343÷2 = 58,996 pairs for the first round of search); we then aggregated the incidence time series of the city pair with the highest correlation coefficient if it exceeded a pre-specified threshold, one pair at a time, and treated the combined city pair as a cluster ‘city’ for the next search. This process was repeated until no more city/cluster pair had a correlation coefficient exceeding the threshold.

For both HCA procedures, city pairs with a correlation coefficient (*r*) above 0.85, an arbitrary threshold, were identified as within the same cluster. As a sensitivity analysis, we also tested clusters identified using thresholds of 0.80 and 0.90. In addition, we visually inspected the epidemic time series for cities within each identified cluster to ensure the quality of the HCA. To reduce any artificial correlation due to concurrent low incidence following the nationwide SIA in September 2010, we restricted this analysis to January 2005 through August 2010 for both HCA procedures.

### Identification of population characteristics associated with an increased burden of measles infection

To examine the association between the burden of measles infection and population variables, we computed the Spearman’s rank correlation coefficient between the cumulative measles incidence rate over the entire study period (i.e. 2005–2014) and each of the population variables (see section “Demographic, population mobility, and socioeconomic Dataset”) among all 344 cities. We used the Spearman’s rank correlation coefficient (*ρ*), as the relationship between measles burden and population variable is nonlinear. To reduce the false positive rate in multiple comparison, we adjusted the *p*-values for raw correlations identified as significant at the 0.05 level, using the Holm-Bonferroni method [38].

### Identification of differences in population characteristics associated with cities in cross-regional v. regional clusters

China is presently comprised of 344 cities in 31 provinces, which are further apportioned among six regions (S1 Fig). To test whether there were outstanding demographic or socio-economic characteristics among cross-regional city-clusters compared to regional city-clusters, we pooled cities identified as part of cross-regional clusters by the HCA (i.e. clusters that include cities from two or more regions) and those in regional clusters (i.e. clusters that include only cities from the same region), respectively, and computed the Spearman’s rank correlation coefficient for these two categories.

This analysis identified two key correlates—the net flux of migrants (i.e. the difference between total and local population size) and economic development—for cities in cross-regional clusters, and another two—the percentage of minorities and natural growth rate—for cities in regional clusters. We hypothesize that these two variable pairs, respectively, define different characteristics among cities in cross-regional v. regional clusters. To test this hypothesis, we used bootstrapping to examine whether the preponderance of these two variable pairs, respectively, would occur by chance alone. Specifically, two sets of 10,000 random samples with matching spatial structure were drawn from the whole 344-city dataset, one with 67 cities in each sample for comparison with cross-regional clusters, and one with 25 cities in each sample for comparison with regional clusters. For instance, a bootstrap sample matching the cross-regional clusters would include 12 subsamples, each with the same numbers of cities drawn from the same regions as in the 12 cross-regional clusters. This test thus controlled for spatial correlation among neighboring cities (for both measles burden and population characteristics). For each set of random samples, we examined 1) the joint distribution of the net flux of migrants and per capita GDP (an indicator of economic development) and 2) the joint distribution of the percentage of minorities and natural growth rate. We used the portion of cities falling in the first quadrant and the third quadrant of the variable-pair plane, respectively, as a proxy characteristic for the joint distribution; for instance, for the plane of net flux of migrants and per capita GDP, those in the 1^st^ quadrant have both variables higher than the national averages, while those in the 3^rd^ quadrant have both variables lower than the national averages. We computed the p-value for the observed (either the cross-regional or regional cluster) based on its location, i.e. percentile, among the distribution of the bootstrapped samples.

## Results

### Three phases of transmission during 2005–2014

**Fig 1:**
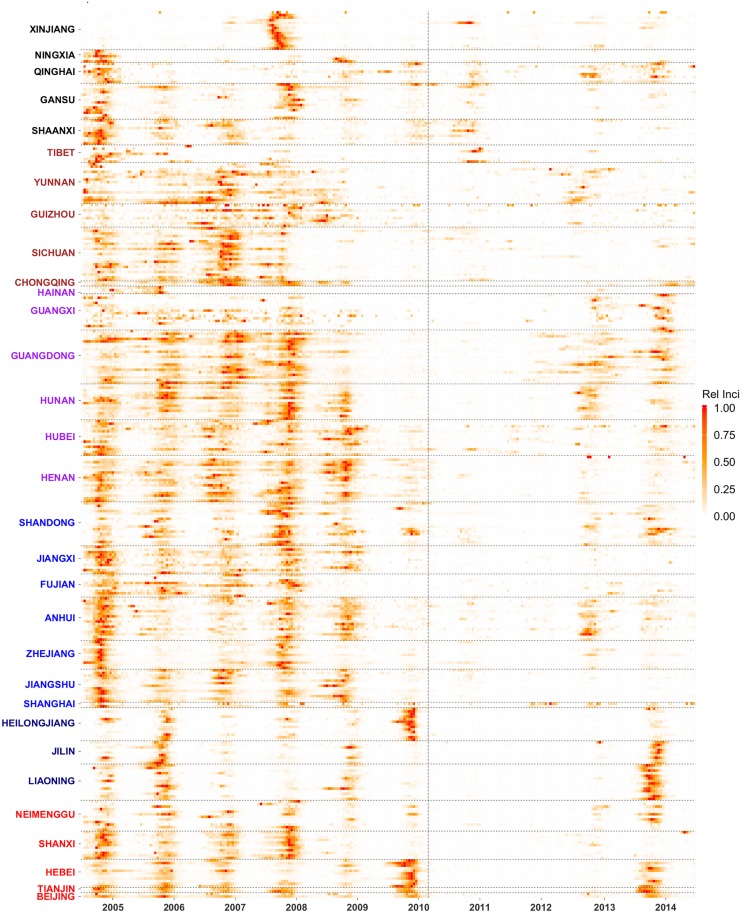
Biweekly measles incidence in each of the 344 cities in China during 2005–2014. Incidence was normalized by dividing the city specific maximum. Cities are organized by province (shown on the y-axis) and region (province labels are color coded for the six regions in China: North in red, Northeast in navy blue, East in blue, South Central in purple, Southwest in brown, and Northwest in black).

A total of 652,852 confirmed measles cases were reported in China during 2005–2014, of which 46.7% were confirmed by laboratory test and the rest by clinical diagnosis. Fig 1 shows the biweekly incidence, relative to the city-specific maximum, for each of the 344 cities in 31 provinces, six regions (i.e. North, Northeast, East, South Central, Southwest and Northwest; S1 Fig) during 2005–2014. This ten-year period can be roughly divided into three phases of transmission: (1) January 2005 to August 2010, a phase with annual outbreaks; (2) September 2010 to December 2012, a lull phase following the nationwide SIA; and (3) January 2013 to December 2014, resurgence of incidence.

During the first phase (i.e. before September 2010), measles recurred regularly each year in the majority of cities, except for those in Northwest China (i.e. Shaanxi, Gansu, Qinghai, Ningxia, and Xinjiang), Tibet and Hainan. The nationwide SIA effectively increased the vaccination coverage, which in turn halted transmission in most cities. During September 2010 to December 2012, tallied over all cities and bi-week periods, measles cases were only recorded 20% of the time (compared to 56% during January 2005 to August 2010). However, it is evident that some cities in Xinjiang, Qinghai, Gansu, Shaanxi and Tibet still saw substantial numbers of cases (orange and red colors in 2011 and 2012 in Fig 1), suggesting that the SIA was less effective in those localities. Beginning in early 2013, measles reappeared in a number of locations, including cities in the Western provinces Yunnan and Qinghai, as well as South Central China (in particular, Guangdong and Hunan) and East China (in particular, Anhui and Shandong). By 2014, this reestablishment has expanded to more cities in Northeast and North China.

### Higher incidence and persistency in mega-cities v. higher incidence rates in inland regions

**Fig 2:**
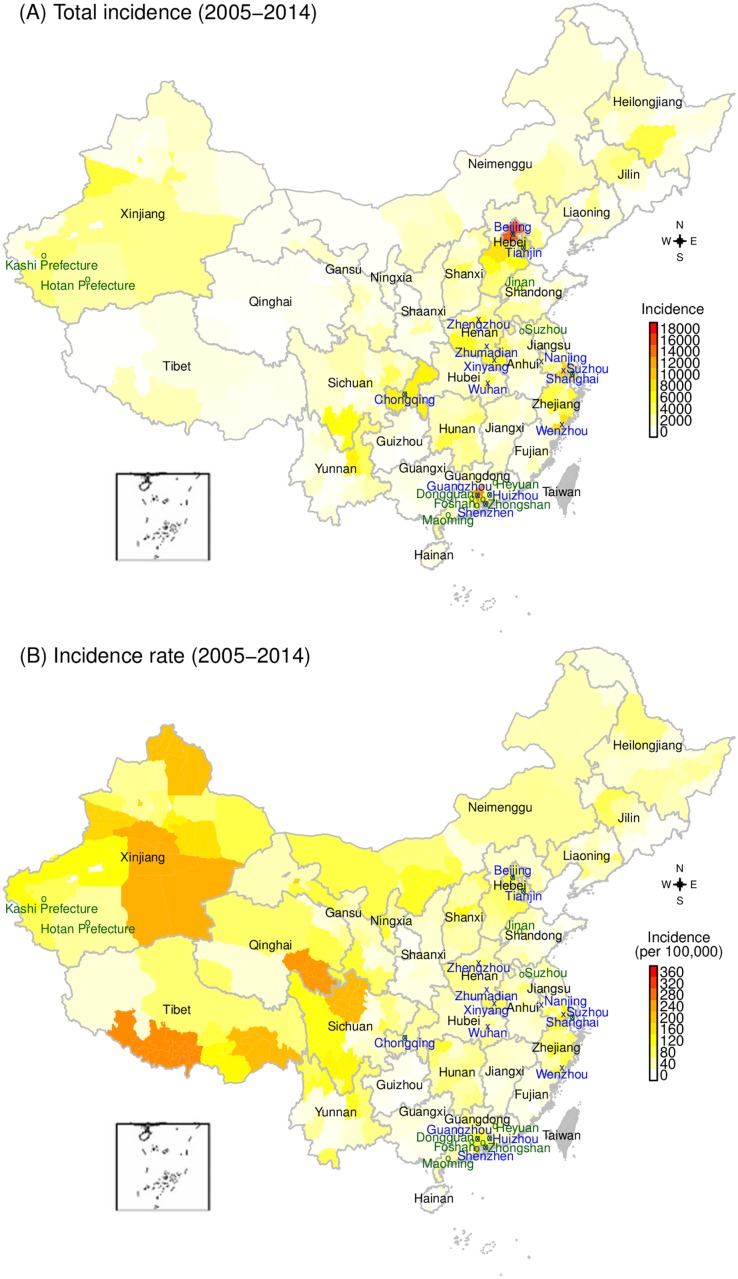
Geographical distribution of measles during 2005–2014. (A) Total incidence by city and (B) Incidence rate by city. Cities labeled in blue and shown by an ‘x’ experienced endemic transmission during January 2005–Auguest 2010; those labeled in green and shown by an ‘o’ experienced endemic transmission during January 2013–December 2014; those indicated by both an ‘x’ and an ‘o’ experienced endemic transmission in both phases.

Not surprisingly, cities with larger populations, mostly located in eastern China (S1 Fig), tended to report larger numbers of cases (Fig 2A). These cities also experienced the most persistent transmission. During the first phase (i.e. before the nationwide SIA in September 2010), there were 14 cities with endemic measles transmission (i.e. reporting cases in all months). Most of these cities were highly industrialized, populous cities located in eastern China, including Beijing, Tianjin, Suzhou, Nanjing, Shanghai, Wenzhou, Guangzhou, Shenzhen, and Huizhou (Fig 2A). The rest were located in three less developed inland provinces (Henan, Hubei, and Chongqing). During the third phase (i.e. January 2013–December 2014), there were 16 endemic cities (Fig 2A and S4 Fig). Of these 16 cities, seven (Beijing, Tianjin, Shanghai, Chongqing, Guangzhou, Shenzhen and Huizhou) also had endemic measles during the first phase, and eight were located in Guangdong province, an industrialized southeastern province attracting the largest number of migrant workers [39,40]. We revisit this connection between measles endemicity and industrialization in subsequent sections.

When examined on a per capita basis (incidence per 100,000 people), a completely different picture emerges (Fig 2B). Cities in the inland most provinces, i.e. Xinjiang, Tibet, Qinghai, and Sichuan, had the highest measles incidence rates. These inland western regions consistently reported higher incidence rates in all three phases of transmission during 2005–2014 (S2–S4 Figs); this difference was particularly evident during September 2010–December 2012 when measles incidence was at its lowest levels in China (S3 Fig). This pattern indicates that, while the total numbers of cases were low, the burden of measles infection in these regions was much higher than the rest of China, and that these regions likely served as refuges for measles when transmission was halted elsewhere following the 2010 nationwide SIA.

### Measles epidemic clusters

**Fig 3:**
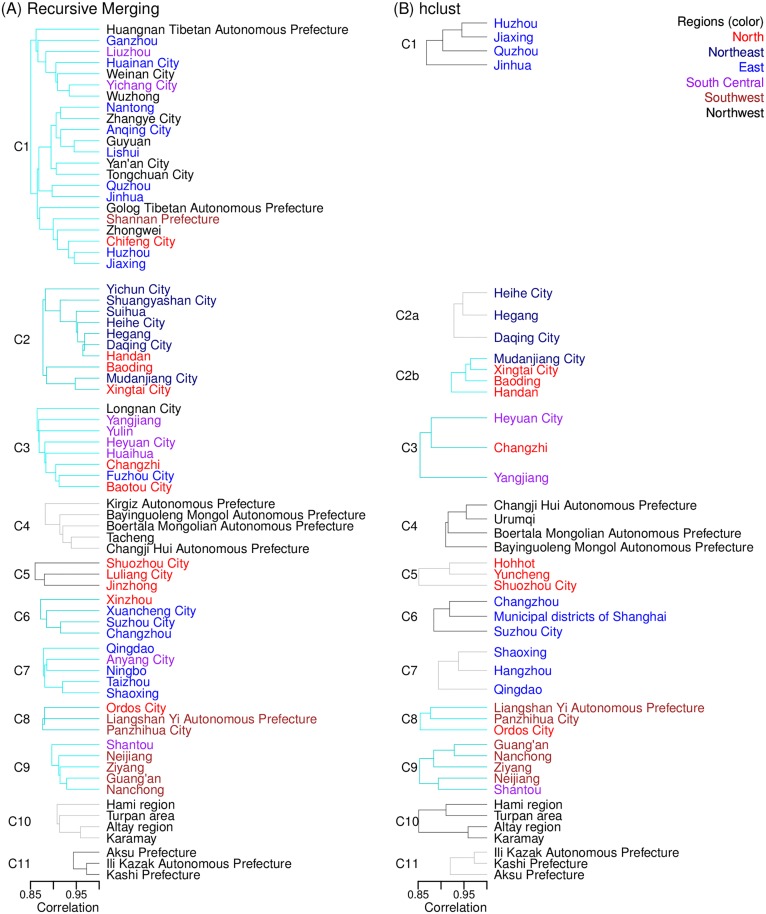
Measles epidemic clusters, identified using the recursive merging algorithm (A) and hierarchical clustering analysis with the complete linkage method (B). For both methods, cities with a Pearson correlation coefficient *r*≥0.85 (shown on the x-axis) were identified as within the same cluster. City-clusters spanning multiple regions are connected with cyan lines while those within the same region are connected with grey lines. Clusters, identified by either method, that include identical or overlapping cities are assigned the same cluster number. Only those with three or more cities are included here; a complete list of clusters is shown in S1 Table.

Given the geospatial heterogeneity in measles burden, it is of interest to see whether there existed a characteristic spatial pattern in the measles outbreaks. In particular, were there epidemic clusters in different regions that would allow for targeted intervention? Further, were there cross-regional clusters that might have facilitated the spread of measles across China? To answer these questions, we performed hierarchical cluster analyses (HCA), using the correlation of measles incidence time series between cities as an indicator of epidemic synchrony and connectivity. The clusters identified by the two HCA methods are either identical (e.g. C7-11 in Fig 3) or overlapping (C1-6 in Fig 3). However, our recursive merging algorithm, by design, captured larger and more cross-regional clusters (e.g., C1 and C2 in Fig 3A v. 3B). For either method, more city clusters were identified when a lower correlation coefficient was used (S1–S3 Tables and Fig 3 and S5 and S6 Figs). Here we report clusters identified using our recursive merging method with *r* = 0.85.

**Fig 4:**
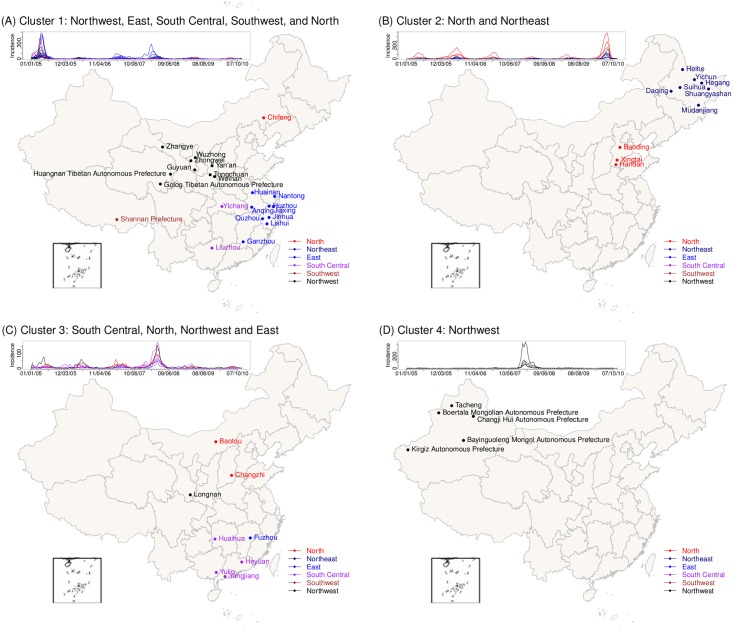
Geographical distributions of city clusters with highly synchronous measles epidemics. (A) Cluster 1 in Fig 3A, including cities located in Northwest, East, South Central, Southwest and North China; (B) Cluster 2 in Fig 3A, including cities located in North and Northeast regions; (C) Cluster 3 in Fig 3A, including cities located in South Central, North, Northwest and East; and (D) Cluster 4 in Fig 3A, including 5 cities in Northwest China.

Using the recursive merging HCA method, we identified 21 epidemic clusters (S1 Table). Fig 4 shows four of the largest clusters. As shown by the inserted time series in Fig 4, epidemic cycles were in concert within the same cluster but differed substantially by cluster. Nine of the 21 clusters were regional. These regional clusters tended to be small (only 2–5 cities) and most cities were located in inland and remote regions (e.g. Fig 4D). The close vicinity of cities within each cluster may explain the epidemic synchrony, while their remoteness may explain the isolation and disconnection with cities outside the region.

The remaining twelve clusters were cross-regional. These clusters tended to include more cities than the regional clusters (2–22 cities). In particular, the largest cluster included 22 cities located in five of the six regions (C1 in Figs 3A and 4A); this cluster captured the connection between the developed East- (9 cities) and underdeveloped Northwest China (9 cities). Likewise, Clusters 3, 6, 7, and 9 (Figs 3A and 4C) likely reflected connections between the underdeveloped inland North and West and the more developed East and South Central regions. These connections suggest that disparity in economic development may be an underlying driver for the synchronous epidemics observed in these cross-regional clusters (we revisit this in the next section).

Interestingly, only two of the 14 cities with endemic measles were captured in any of the clusters: Suzhou City (in C6 of Fig 3A) and Shanghai (in C6 of Fig 3B). Even when a lower threshold was used (*r* = 0.8), only 6 endemic cities (i.e. Shanghai, Nanjing, Suzhou, Guangzhou, Huizhou and Chongqing) were identified among clusters.

### Characteristics associated with high measles burden

To identify key population characteristics that may be associated with measles burden, we examined the correlation between cumulative measles incidence rate and 23 variables related to 1) demographics, including population size and density, age structure, birth and death rates, ethnicity composition, and education level; 2) population mobility; and 3) socioeconomics, including gross domestic product (GDP), GDP composition, and urbanization. Table 1 shows the distributions of the 23 variables along with their Spearman’s rank correlation with cumulative measles incidence rate.

**Table 1:** Distribution of population variables and their correlations with measles burden. Variable names are listed in the first column along with the number of cities (n) included; the minimum (Min), median, mean, and maximum (Max) value for each variable are shown in the corresponding columns. Spearman’s rank correlation coefficient (ρ), p-value, and adjusted p-value using the Holm-Bonferroni method between each variable and the cumulative measles incidence rate (2005–2014) in cities are shown in the last three columns; variables correlated significantly with cumulative measles incidence rate at the 0.05 level after adjusted using the Holm-Bonferroni method were bolded.

Variables	Min	Median	Mean	Max	ρ	p-value	Adjusted p-value
**Percentage of 65+ yr olds (%; n = 344)**	**1.79**	**8.73**	**8.68**	**17.9**	**-0.41**	**0**	**0**
**Difference between total and local population relative to total population (%;n = 344)**	**-45.6**	**-2.95**	**-2.13**	**77.44**	**0.35**	**2.48e-11**	**2.48e-10**
**Death Rate (‰; n = 344)**	**0.85**	**5.66**	**5.59**	**15.08**	**-0.30**	**1.42e-8**	**1.28e-7**
**Minorities (%; n = 344)**	**0.0087**	**2.05**	**15.5**	**97.19**	**0.28**	**2.04e-7**	**1.63e-6**
**Share of GDP from agriculture and agriculture-related industries (%; n = 289)**	**0.07**	**12.73**	**13.41**	**44.78**	**-0.28**	**1.84e-6**	**1.29e-5**
**Percentage of population migrated from other province among total (%; n = 344)**	**0.11**	**1.70**	**4.84**	**64.87**	**0.23**	**2.48e-5**	**0.00015**
**Local population (in thousands; n = 344)**	**89.74**	**3335**	**3888**	**17540**	**-0.18**	**9.16e-4**	**0.0046**
**Percentage of 15–64 yr olds (%; n = 344)**	**61.22**	**74.28**	**74.08**	**89.43**	**0.17**	**2.16e-3**	**0.0086**
**Percentage of population migrated from counties in the same province among total (%; n = 344)**	**0.39**	**3.94**	**5.76**	**32.48**	**0.14**	**9.49e-3**	**0.029**
**Share of GDP from service industries (%; n = 289)**	**9.76**	**34.77**	**35.67**	**75.51**	**0.15**	**0.01**	**0.029**
**Per capita GDP (CNY; n = 340)**	**5181**	**25330**	**31500**	**175100**	**0.13**	**0.01**	**0.029**
Total population (in thousands; n = 344)	95.46	3185	3874	22320	-0.10	0.07	NA
Average year of education (n = 344)	3.46	8.82	8.73	11.77	0.09	0.08	NA
Percentage of non-agriculture population among total (%; n = 344)	6.44	25.67	29.65	86.21	0.09	0.08	NA
Population density (/km^2^; n = 344)	0.29	272.6	426.4	6262	-0.09	0.09	NA
Natural growth rate (‰; n = 344)	-4.25	5.11	5.13	18.14	0.08	0.14	NA
Percentage of illiterate among 15+ yr olds (%; n = 344)	0.54	4.44	6.14	46.32	-0.06	0.26	NA
Share of GDP from manufacturing industries (%; n = 289)	17.12	51.24	50.93	89.75	0.06	0.34	NA
Percentage of urban population (%; n = 344)	12.69	44.11	47.46	100	0.04	0.51	NA
Percentage of rural population (%; n = 344)	0	55.89	52.54	87.31	-0.04	0.51	NA
Birth rate (‰; n = 344)	2.91	10.48	10.72	23.22	-0.03	0.59	NA
Total GDP (CNY; n = 340)	1.85e9	7.36e10	1.30e11	1.70e12	0.01	0.88	NA
Percentage of 0–14 yr olds (%; n = 344)	8.15	16.81	17.24	31.86	-0.01	0.92	NA

Among all 344 cities, variables that correlated significantly with cumulative measles incidence rate spanned all three categories (i.e. demographics, population mobility, and socioeconomics). Among demographic variables, the percentage of 65+ yr olds (*ρ* = −0.41, *p* = 0), death rate (*ρ* = −3.0, *p* = 1.28e-7), percentage of minorities (*ρ* = 0.28, *p* = 1.63e-6), local population size (*ρ* = −0.18, *p* = 0.0046), and percentage of 15–64 yr olds (*ρ* = 0.17, *p* = 0.0086) were most statistically significant. The Spearman’s rank coefficients (*ρ*) for these variables indicate that cities with larger young adult populations (15–64 yr olds), larger minority populations, lower death rates, and smaller local population sizes tended to have higher measles burdens.

Among population mobility variables, the difference between total and local population relative to total population (i.e. net influx of migrants; *ρ* = 0.35, *p* = 2.48e-10), percentage of population migrated from other provinces (*ρ* = 0.23, *p* = 0.00015), and percentage of population migrated from counties in the same province (*ρ* = 0.14, *p* = 0.029) were most statistically significant. These correlations indicate population migration was associated with measles outbreaks, with cities attracting larger numbers of migrants experiencing higher measles burdens. In addition, the highly significant correlation with long-range migration (i.e. those from outside provinces) suggests that population migration might link cities identified in the aforementioned cross-regional clusters.

Among variables related to socioeconomic development, the share of GDP from agriculture and agriculture-related industries (*ρ* = −0.28, *p* = 1.29e-5), the share of GDP from service industries (*ρ* = 0.15, *p* = 0.029), and per capita GDP (*ρ* = 0.13, *p* = 0.029) were most statistically significant. These three correlations all indicate that economically more developed cities were more likely to experience higher measles burdens. This observation may appear counterintuitive, as more developed cities are more likely to have better public health infrastructure (in particular, better immunization programs) and one would expect a lower risk of measles transmission; however, developed cities are also those attracting the largest numbers of migrant workers, which, as shown above, were places with higher measles incidence rates. Taken together, the association of higher measles incidence rates with higher migration rates and greater economic development points to a connection with population migration driven by the disparity in economic development among regions in China. Such migration may have facilitated the spread of measles in industrialized cities and linked more developed regions in East China with less developed inland regions. This finding is consistent with the cross-regional clusters identified in Figs 3 and 4, and with the finding that a large number of endemic cities are highly industrialized cities that attract large numbers of migrant workers.

### Characteristics associated with cross-regional v. regional clusters

**Fig 5:**
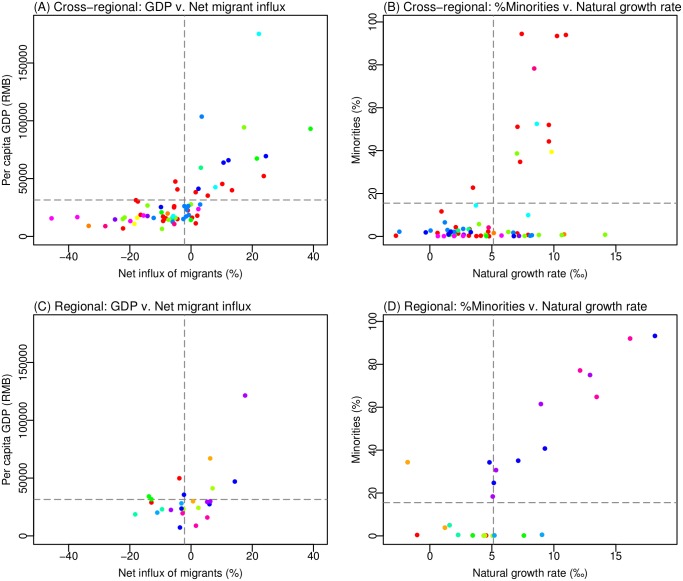
Difference in key population characteristics among cross-regional and regional city-clusters. Scatter plots of per capita GDP and net influx of migrants among cities in cross-regional clusters (A) and those in regional clusters (C). Scatter plots of minority percentage and natural growth rate (i.e. birth rate minus death rate) among cities in cross-regional clusters (B) and those in regional clusters (D). Points in the same color denote cities from the same cluster; dashed grey lines indicate the national averages for the corresponding variables (i.e. per capita GDP, net flux of migrants, rate of minorities and natural growth rate).

Different demographic and socioeconomic characteristics were associated with the cities identified in the cross-regional v. regional clusters. As shown in Table 2, for cities in the cross-regional clusters, variables correlated significantly with measles incidence rate were two population mobility indicators (i.e. net influx of migrants and migrants from outside provinces) and one economic indicator (i.e. share of GDP from service industries). In comparison, for cities in regional clusters, high measles incidence rates were significantly associated with 13 variables, broadly related to 5 demographic and socioeconomic features: 1) high percentages of minorities (*ρ* = 0.75, *p* = 0.00032); 2) high rates of population growth, including low percentages of elderly (*ρ* = −0.86, *p* = 3.3e-5) and high percentages of children (*ρ* = 0.61, *p* = 0.011), low death rates (*ρ* = −0.78, *p* = 8.7e-5), high natural growth rates (*ρ* = 0.78, *p* = 9.1e-5), and high birth rates (*ρ* = 0.48, *p* = 0.049); 3) small population size and density, including small local (*ρ* = −0.62, *p* = 0.0098) and total (*ρ* = −0.61, *p* = 0.011) population size and low population density (*ρ* = −0.85, *p* = 3.3e-5); 4) slow socioeconomic development, as indicated by the low total GDP (*ρ* = −0.61, *p* = 0.011); and 5) high population mobility, including high net influx of migrants (*ρ* = 0.52, *p* = 0.032) and large numbers of migrants from outside provinces (*ρ* = 0.6, *p* = 0.011). The larger number of significant correlates identified for regional clusters is not surprising, as cities in the same region tend to have more aligned measles epidemic dynamics, due to their closer spatial vicinity, as well as more similar demographic and socioeconomic characteristics. Further, we note that natural growth rates (a combination of birth rate and death rate) and percentages of minorities are highly positively correlated, as shown in Fig 5D.

**Table 2:** Correlation between population variables and measles burden among cities in cross-regional clusters, regional clusters, and all cities. Variable names are listed in the first column. Numbers in the last three columns show the Spearman’s rank correlation coefficient (ρ) and adjusted *p*-value (in parentheses) between each variable and the cumulative measles incidence rate (2005–2014) for each category (i.e. cross-regional, regional, or all cities). The Holm-Bonferroni method was used to adjust the *p*-values, however, only applied to those with unadjusted values below 0.05; bold numbers indicate significant correlation at the 0.05 level.

Variable	Cross-regional (n = 67)	Regional (n = 25)	All cities (n = 344)
Difference between total and local population relative to total population	**0.39 (0.0036)**	**0.52 (0.032)**	**0.35 (2.5e-10)**
Percentage of population migrated from other provinces among total	**0.34 (0.011)**	**0.6 (0.011)**	**0.23 (0.00015)**
Share of GDP from service industries (%)	**0.34 (0.011)**	-0.45 (0.1)	**0.15 (0.028)**
Minorities (%)	0.24 (0.051)	**0.75 (0.00032)**	**0.28 (1.6e-6)**
Share of GDP from agriculture and agriculture-related industries (%)	-0.24 (0.058)	-0.45 (0.11)	**-0.28 (1.3e-5)**
Percentage of illiterate among 15+ yr olds (%)	0.23 (0.064)	**-0.7 (0.0013)**	-0.06 (0.26)
Percentage of 65+ yr olds (%)	-0.22 (0.072)	**-0.86 (3.3e-5)**	**-0.41 (0)**
Per capita GDP (CNY)	0.22 (0.076)	-0.26 (0.2)	**0.13 (0.028)**
Percentage of population migrated from counties in the same province among total	0.18 (0.14)	-0.24 (0.25)	**0.14 (0.028)**
Local population	-0.15 (0.22)	**-0.62 (0.0098)**	**-0.18 (0.0046)**
Death rate (‰)	-0.14 (0.25)	**-0.78 (8.7e-5)**	**-0.3 (1.3e-7)**
Average year of education	-0.13 (0.29)	0.39 (0.055)	0.09 (0.079)
Percentage of 15–64 yr olds (%)	0.1 (0.43)	-0.09 (0.67)	**0.17 (0.0086)**
Total population	-0.08 (0.51)	**-0.61 (0.011)**	-0.1 (0.069)
Percentage of non-agriculture population among total (%)	-0.08 (0.53)	0.14 (0.51)	0.09 (0.084)
Total GDP (CNY)	0.06 (0.62)	**-0.61 (0.011)**	0.01 (0.88)
Natural growth rate (‰)	0.05 (0.71)	**0.78 (9.1e-5)**	0.08 (0.14)
Share of GDP from manufacturing industries (%)	0.04 (0.76)	0.42 (0.13)	0.06 (0.34)
Percentage of urban population (%)	0.01 (0.93)	-0.43 (0.07)	0.04 (0.51)
Percentage of rural population (%)	-0.01 (0.93)	0.43 (0.07)	-0.04 (0.51)
Population density (/km^2^)	0.01 (0.94)	**-0.85 (3.3e-5)**	-0.09 (0.085)
Birth rate (‰)	-0.01 (0.97)	**0.48 (0.049)**	-0.03 (0.59)
Percentage of 0–14 yr olds (%)	0 (0.99)	0.61 (0.011)	-0.01 (0.92)

The joint distribution of net flux of migrants and per capita GDP and that of percentages of minorities and natural growth rates showed different characteristics for the two types of clusters (Fig 5). For cities in cross-regional clusters, the distributions of per capita GDP and net flux of migrants were highly dispersed—i.e. cities were from either highly developed or highly underdeveloped regions—and highly correlated, falling in the 1^st^ and 3^rd^ quadrants among all cities in China (Fig 5A). In comparison, for cities in regional clusters, the joint distribution was narrower—i.e. cities were more clustered around the mean for economic development and migration—and less correlated (Fig 5C). On the other hand, the distributions of minority population and natural growth rate (i.e. birth rate minus death rate) were more dispersed and highly correlated for cities in regional clusters, falling in the 1^st^ and 3^rd^ quadrants (Fig 5D), but less so for those in cross-regional clusters (Fig 5B).

Based on the above findings, we hypothesized that different mechanisms underlie the connections among cities in cross-regional v. regional clusters. For the former, internal worker migration (as indicated by the net flux of migrants), in response to uneven economic development, connects cities in cross-regional clusters and contributes to the epidemic synchrony among those cities. For the regional clusters, in addition to spatial vicinity, high percentages of minorities and natural growth rates favor similar fluctuation in susceptibility and thus epidemic synchrony among those cities. Indeed, bootstrap sampling analysis (Table 3), controlled for spatial structure, showed that the cross-regional clusters included a larger portion of cities with both low levels of net migrant-flux (i.e. more emigrants) and per-capita GDP (*p* = 0.011) and a larger portion of cities with both low levels of minorities and natural growth rates (*p* = 0.025), suggesting that migration from economically less-developed cities to more developed regions may have linked cities across regions and facilitated cross-regional measles transmission. In contrast, the regional-clusters included a *smaller* portion of cities with both high levels of net migrant-flux and per-capita GDP (*p* = 0.060) and a larger portion of cities with both high levels of minorities and natural growth rates (*p* = 0.074), suggesting that more cities in regional clusters were minority-dominant and less connected to outside regions (via migration).

**Table 3:** Characteristic indicators for the connection among cities in cross-regional v. regional clusters. Two sets of 10,000 random samples were drawn from the whole 344-city dataset without replacement, one with 67 cities each for comparison with cross-regional clusters, and one with 25 cities each for comparison with regional clusters. The portions of cities lying in the 1^st^ or 3^rd^ quadrant of 1) the joint distribution of the net influx of migrants and per capita GDP and 2) the joint distribution of the percentage of minorities and natural growth rate were used as proxy characteristics for the joint distributions. Numbers are the observed portion v. the mean of the bootstrap samples and 95% CI in the square brackets; cells in bold indicates that the observed characteristic for either the cross-regional or the regional clusters is significantly different from samples at random at the 0.1 level.

Variables	Distribution characteristics	Cross-regional clusters v. at random	Regional clusters v. at random
Net flux of migrants & per-capita GDP	Portion in 1st Quadrant: both at high levels	0.24 v 0.30 [0.21, 0.40] (*p* = 0.114)	**0.16 v 0.27 [0.12, 0.44] (*p* = 0.06)**
	Portion in 3rd Quadrant: both at low levels	**0.55 v 0.43 [0.32, 0.53] (*p* = 0.011)**	0.40 v 0.42 [0.25, 0.60] (*p* = 0.989)
%minorities & natural growth rate	Portion in 1st Quadrant: both at high levels	0.16 v 0.19 [0.11, 0.26] (*p* = 0.29)	**0.40 v 0.28 [0.14, 0.43] (*p* = 0.074)**
	Portion in 3rd Quadrant: both at low levels	**0.57 v 0.46 [0.36, 0.57] (*p* = 0.025)**	0.36 v 0.43 [0.25, 0.60] (*p* = 0.2)

## Discussion

While reported vaccination rates have been above 90% during the last decade, China continues to document a substantial number of measles cases. Using prefectural city level incidence and demographic data, our study maps the geospatial distribution of measles incidence and incidence rates across China during the past decade. Further, using two approaches—hierarchical clustering analysis and Spearman’s rank correlation—our study reveals the key population characteristics associated with cities that experienced a higher burden of measles infection as well as differing characteristics associated with cross-regional v. regional epidemic clusters.

Among the 344 Chinese cities, large population and economic centers not only reported the largest numbers of measles cases, but also recorded cases more often, i.e. had more bi-weeks with nonzero reported cases. We identified 14 cities with endemic measles transmission during January 2005 –August 2010, of which 10 (Beijing, Tianjin, Shanghai, Suzhou, Nanjing, Wenzhou, Guangzhou, Shenzhen, Huizhou and Wuhan) are among the most economically developed [per capita GDP: 74252±23536 Chinese Yuan (CNY) v. 31503±23489 over all cities]. Even when evaluated on a per capita basis, these cities still had incidence rates significantly higher than the national average (82.5±37.8 v. 46.3± 41.0 per 100,000 over 2005–2010; *p* = 0.0038). Our analyses of measles epidemic clusters and their population characteristics consistently identify internal migrant movement as a factor contributing to the higher measles burden in these industrial cities. Due to disparities in economic development, each year, over 100 million Chinese leave their hometowns, predominantly in inland regions, to work in big cities, primarily located in eastern China (e.g. Beijing, Tianjin, Shanghai, Jiangsu, Zhejiang, and Guangdong)[41]. This massive population movement likely replenished the measles susceptible pools in these industrial cities, fomented outbreaks with higher incidence rates and larger numbers of cases, and contributed to the persistence of measles in those cities. Indeed, recent outbreak investigations indicated that migrant workers were disproportionally infected in big cities [42–44].

Further, our analyses indicate that internal worker migration likely linked measles outbreaks in cities across China. We identified 12 cross-regional epidemic clusters with highly synchronous measles outbreaks; in particular, six of the clusters linked inland regions with eastern China. Among cities identified in these cross-regional clusters, long-distance migration rate was significantly correlated with measles incidence rate (*ρ* = 0.34, *p* = 0.011, Table 2). In addition, while information on the origin of migrants was not available, using the difference in total and local population as a proxy for net influx of migrant, we showed that cities in the cross-regional clusters had either large numbers of immigrants (i.e. positive net influx) or large numbers of emigrants (i.e. negative net influx, Fig 5A). It is likely that megacities served as mixing grounds for migrant populations from different regions in China; and the seasonal return of those migrant workers to their hometowns might have facilitated the reintroduction of measles to rural communities. This putative mechanism is also consistent with the timing of the spring outbreaks observed over our study period [9], as worker migration typically surges round the Chinese New Year (between late January and late February). Further investigation into this potential source-sink interaction and feedback as well as its role in cross-regional transmission and persistence of measles in China is warranted.

Remarkably, the identified clusters did not include most of the industrial cities that experienced endemic measles prior to the 2010 nationwide SIA. Mega-industrial cities tend to attract migrant workers from a larger number of locations across China and as a result, the measles epidemic cycle in each city is likely influenced by multiple sources and does not resemble that of one particular source. In addition, those endemic cities did not have synchronous epidemics with one another, and were likely each a transmission hub. For instance, the three endemic cities in Guangdong—Guangzhou, Shenzhen and Huizhou—had asynchronous epidemic cycles despite their close vicinity and high connectivity (*r* = 0.39, 0.44, and 0.57, well below the *r* = 0.85 HCA threshold). Such asynchrony in endemic cities also creates challenges for eliminating measles in China.

Another key population characteristic is the association of high measles burden with a high percentage of minorities, in particular, among cities identified within regional clusters (Table 2). Minority-dominated cities tend to be located in inland regions and are less connected to outside regions, which likely explains the regional clustering. The reason for the higher measles burden is likely two-fold: 1) immunization programs were likely less stringently or effectively implemented in these cities and 2) as shown in Fig 5D, these minority-dominated communities tended to have higher birth rates. As a result, these populations were likely to have larger fractions of susceptible persons and to be vulnerable to larger outbreaks should measles infection be introduced from an outside community. These characteristics may have supported the rare, larger outbreaks recorded in some cities, e.g. those in Xinjiang province (Fig 4D). Enhanced vaccination implementation is thus needed in these regions with specific targeting of minority populations.

On a related note, high birth rates were only found to be associated with high measles incidence rates among cities in regional clusters (*ρ* = 0.48, *p* = 0.049, after adjusting for other correlates; Table 2), but not among all 344 cities (*ρ* = −0.03, *p* = 0.59) nor among those in cross-regional clusters (*ρ* = −0.01, *p* = 0.97). Birth rate is an important factor in measles epidemiology, as it affects the recruitment rate of susceptible individuals and in turn epidemic frequency [13,45,46]. The absence of a significant rank correlation over the whole 344-city dataset may be partly due to China’s one-child policy implemented during 1979–2015, which led to low nationwide birth rates [47,48]. It is interesting to note that minorities were allowed to have 2–3 children under China’s one-child policy [47], and many of the cities with highest incidence rates had large minority populations (Fig 5D). As China has adopted its new two-child policy in 2016 [49], birth rates may increase in other inland regions. New baby booms in these regions may create further challenges for the elimination of measles.

We recognize a number of caveats in this study. First, we do not have city-level measles immunization coverage data to assess the relationship between measles burden and measles vaccination rates. In addition to the synchronized nationwide SIAs in 2010, multiple subnational SIAs (typically at the provincial level) were conducted prior to 2010 [50], which might have increased the epidemic synchrony among cities undergoing the same SIAs. However, the impact of such forced synchrony is likely small. The majority of cities in China experienced outbreaks annually prior to 2010 (Fig 1), suggesting that those subnational SIAs were less effective. In addition, most cross-regional clusters identified here included only a small fraction of cities from each province (S1 Table). Should province-specific SIAs be the determinant of epidemic synchrony, more cities within the same province would have been included in the same cluster. Nevertheless, future work should investigate the impact of subnational SIAs on epidemic dynamics when city-level vaccination data become available.

Second, while the CISDCP surveillance system includes nearly all medical institutions in China [28,51], underreporting may still exist and reporting rates may vary by city and time. In particular, rural areas likely have less developed health facilities and higher underreporting rates [52]; reporting rates were also likely lower in 2012 for all cities, as China strived to meet the measles elimination goal set for that year. Third, we do not have information on the ethnicity of each measles case. While our analyses indicate significantly higher incidence rates in minority-dominated cities, further studies are warranted to investigate whether these infections indeed occurred within minority groups. Similarly, we do not have information on migration between specific cities or the occupation of each measles incident case (i.e. whether a case is a migrant worker); as such, our analyses are ecological (i.e. based on population level variables). Further investigations with such detailed information are needed to establish a more direct connection between internal worker migration and the transmission of measles across cities. Finally, we focused on the geospatial characteristics of measles outbreaks and sidestepped other key issues related to measles transmission dynamics, such as the seasonality and age profile of measles cases. Studies to investigate these issues are underway.

In summary, our analysis of the geography of measles demonstrates that multiple endemic cities co-exist in China, and that these cities are predominantly industrial cities with large numbers of migrant workers, whose movement likely facilitates the spread of measles across regions in China. In addition, cities with large minority communities and inland, underdeveloped cities likely have lower vaccination rates and are more vulnerable to the resurgence of measles. Measles has reappeared in many cities in China since 2013. Our findings suggest that elimination of measles remains challenging in China due to the heterogeneity in vaccination implementation and complex connections among regions.

## Supporting information

S1 Table City clusters with synchronous epidemic cycles. Cities with a Pearson correlation coefficient *r*≥0.85 were identified as within the same cluster. The 1^st^ column shows the cluster id number, the 2^nd^ shows the total number of cities included in each cluster, the 3^rd^ lists the cities in each cluster, the 4^th^ lists the province(s) in each cluster, and the 5^th^ lists the region(s) in each cluster. The numbers in the parentheses in the 4^th^ and 5^th^ columns indicate the numbers of cities located in each province or region.
(DOCX)

S2 Table Same as S1 Table but with *r*≥0.80. (DOCX)

S3 Table Same as S1 Table but with *r*≥0.90. (DOCX)

S1 Fig Regions and population density in China. There are six geographical regions in China; provinces in the same region are labeled and outlined in the same color: North in red, Northeast in navy blue, East in blue, South Central in purple, Southwest in brown, and Northwest in black. Prefecture city level population density is shown by color as indicated in the legend.
(TIF)

S2 Fig Cumulative measles incidence rate over the 1^st^ phase of transmission (January 2005–August 2010) by city. Cities labeled in blue and shown by an ‘x’ experienced endemic transmission during this phase.
(TIF)

S3 Fig Cumulative measles incidence rate over the 2^nd^ phase of transmission (September 2010–December 2012) by city. None of the 344 cities experienced endemic transmission during this phase.
(TIF)

S4 Fig Cumulative measles incidence rate over the 3^rd^ phase of transmission (January 2013–December 2014) by city. Cities labeled in green and shown by an ‘o’ experienced endemic transmission during this phase.
(TIF)

S5 Fig Same as in Fig 3 but with *r*≥0.80. Only those with three or more cities are included here; a complete list of clusters is shown in S2 Table.
(PDF)

S6 Fig Same as in Fig 3 but with *r*≥0.90. Only those with three or more cities are included here; a complete list of clusters is shown in S3 Table.
(PDF)
