# Correction: Geospatial characteristics of measles transmission in China during 2005−2014

**DOI:** 10.1371/journal.pcbi.1007144

**Published:** 2019-06-14

**Authors:** Wan Yang, Liang Wen, Shen-Long Li, Kai Chen, Wen-Yi Zhang, Jeffrey Shaman

## Notice of republication

This article was republished on January 16, 2018, to correct information provided in the Data Availability statement. Please download this article again to view the correct version.

## Supporting information

S1 File Republished, corrected article. (PDF)
