# Supplementary material for: Geospatial characteristics of measles transmission in China during 2005−2014
Source: PLoS Comput Biol. 2017 Apr 4;13(4):e1005474. doi: 10.1371/journal.pcbi.1005474 (PMC5395235; doi:10.1371/journal.pcbi.1005474)
Supplement: S1 Table — Cities with a Pearson correlation coefficient r≥0.85 were identified as within the same cluster. The 1st column shows the cluster id number, the 2nd shows the total number of cities included in each cluster, the 3rd lists the cities in each cluster, the 4th lists the province(s) in each cluster, and the 5th lists the region(s) in each cluster. The numbers in the parentheses in the 4th and 5th columns indicate the numbers of cities located in each province or region. (DOCX) [file pcbi.1005474.s001.docx]

**Table S1.** City clusters with synchronous epidemic cycles. Cities with a Pearson correlation coefficient *r*≥0.85 were identified as within the same cluster. The 1^st^ column shows the cluster id number, the 2^nd^ shows the total number of cities included in each cluster, the 3^rd^ lists the cities in each cluster, the 4^th^ lists the province(s) in each cluster, and the 5^th^ lists the region(s) in each cluster. The numbers in the parentheses in the 4^th^ and 5^th^ columns indicate the numbers of cities located in each province or region.

| **No.** | **# cities** | **Cities** | **Province(s)** | **Region(s)** |
| --- | --- | --- | --- | --- |
| 1 | 22 | Chifeng; Nantong; Jiaxing, Huzhou, Jinhua, Quzhou, Lishui; Huainan, Anqing; Ganzhou; Yichang; Liuzhou; Shannan Prefecture; Tongchuan, Weinan, Yan'an; Zhangye; Huangnan Tibetan Autonomous Prefecture, Golog Tibetan Autonomous Prefecture; Wuzhong, Guyuan, Zhongwei | Neimenggu (1); Jiangshu (1), Zhejiang (5), Anhui (2), Jiangxi (1); Hubei (1), Guangxi (1); Tibet (1); Shaanxi (3), Gansu (1), Qinghai (2), Ningxia (3) | North (1); East (9); South Central (2); Southwest (1); Northwest (9) |
| 2 | 10 | Handan, Xingtai, Baoding; Hegang, Shuangyashan, Daqing, Yichun, Mudanjiang, Heihe, Suihua | Hebei (3); Heilongjiang (7) | North (3); Northeast (7) |
| 3 | 8 | Changzhi; Baotou; Fuzhou; Huaihua; Heyuan, Yangjiang; Yulin; Longnan | Shanxi (1), Neimenggu (1); Jiangxi (1); Hunan (1), Guangdong (2), Guangxi (1); Gansu (1) | North (2); East (1); South Central (4); Northwest (1) |
| 4 | 5 | Changji Hui Autonomous Prefecture, Boertala Mongolian Autonomous Prefecture, Bayinguoleng Mongol Autonomous Prefecture, Kirgiz Autonomous Prefecture, Tacheng | Xinjiang (5) | Northwest (5) |
| 5 | 5 | Ningbo, Shaoxing, Taizhou; Qingdao; Anyang | Zhejiang (3), Shandong (1); Henan (1) | East (4); South Central (1) |
| 6 | 5 | Shantou; Neijiang, Nanchong, Guang'an, Ziyang | Guangdong (1); Sichuan (4) | South Central (1); Southwest (4) |
| 7 | 4 | Xinzhou; Changzhou, Suzhou; Xuancheng | Shanxi (1); Jiangshu (2), Anhui (1) | North (1); East (3) |
| 8 | 4 | Karamay, Turpan area, Hami region, Altay region | Xinjiang (4) | Northwest (4) |
| 9 | 3 | Shuozhou, Jinzhong, Luliang | Shanxi (3) | North (3) |
| 10 | 3 | Ordos; Panzhihua, Liangshan Yi Autonomous Prefecture | Neimenggu (1); Sichuan (2) | North (1); Southwest (2) |
| 11 | 3 | Aksu Prefecture, Kashi Prefecture, Ili Kazak Autonomous Prefecture | Xinjiang (3) | Northwest (3) |
| 12 | 2 | Huaian, Yangzhou | Jiangshu (2) | East (2) |
| 13 | 2 | Fuyang; Kaifeng | Anhui (1); Henan (1) | East (1); South Central (1) |
| 14 | 2 | Dandong, Panjin | Liaoning (2) | Northeast (2) |
| 15 | 2 | Southwest Guizhou Buyi and Miao Autonomous Prefecture; Qingyang | Guizhou (1); Gansu (1) | Southwest (1); Northwest (1) |
| 16 | 2 | Xuzhou, Yancheng | Jiangshu (2) | East (2) |
| 17 | 2 | Leshan, Meishan | Sichuan (2) | Southwest (2) |
| 18 | 2 | Tangshan; Qiqihar | Hebei (1); Heilongjiang (1) | North (1); Northeast (1) |
| 19 | 2 | Yueyang; Zhanjiang | Hunan (1), Guangdong (1) | South Central (2) |
| 20 | 2 | Loudi; Dazhou | Hunan (1); Sichuan (1) | South Central (1); Southwest (1) |
| 21 | 2 | Miao and Dong Autonomous Prefecture; Wuwei | Guizhou (1); Gansu (1) | Southwest (1); Northwest (1) |
