# Supplementary material for: Geospatial characteristics of measles transmission in China during 2005−2014
Source: PLoS Comput Biol. 2017 Apr 4;13(4):e1005474. doi: 10.1371/journal.pcbi.1005474 (PMC5395235; doi:10.1371/journal.pcbi.1005474)
Supplement: S2 Table — (DOCX) [file pcbi.1005474.s002.docx]

**Table S2.** City clusters with synchronous epidemic cycles. Cities with a Pearson correlation coefficient *r*≥0.80 were identified as within the same cluster. The 1^st^ column shows the cluster id number, the 2^nd^ shows the total number of cities included in each cluster, the 3^rd^ lists the cities in each cluster, the 4^th^ lists the province(s) in each cluster, and the 5^th^ lists the region(s) in each cluster. The numbers in the parentheses in the 4^th^ and 5^th^ columns indicate the numbers of cities located in each province or region.

| **No.** | **# cities** | **Cities** | **Province(s)** | **Region(s)** |
| --- | --- | --- | --- | --- |
| 1 | 24 | Taiyuan, Datong, Shuozhou, Jinzhong, Yuncheng, Xinzhou, Luliang; Hohhot, Wulanchabu; Municipal districts of Shanghai; Nanjing, Changzhou, Suzhou, Zhenjiang; Hefei, Anqing, Fuyang, Xuancheng; Jiujiang, Ji'an; Kaifeng; Baoji; Lanzhou, Zhangye | Shanxi (7), Neimenggu (2); Shanghai (1), Jiangshu (4), Anhui (4), Jiangxi (2); Henan (1); Shaanxi (1), Gansu (2) | North (9); East (11); South Central (1); Northwest (3) |
| 2 | 22 | Chifeng; Nantong; Jiaxing, Huzhou, Jinhua, Quzhou, Lishui; Huainan; Ganzhou, Shangrao; Yichang; Shannan Prefecture; Xi'an, Tongchuan, Xianyang, Weinan, Yan'an; Huangnan Tibetan Autonomous Prefecture, Golog Tibetan Autonomous Prefecture; Wuzhong, Guyuan, Zhongwei | Neimenggu (1); Jiangshu (1), Zhejiang (5), Anhui (1), Jiangxi (2); Hubei (1); Tibet (1); Shaanxi (5), Qinghai (2), Ningxia (3) | North (1); East (9); South Central (1); Southwest (1); Northwest (10) |
| 3 | 16 | Changzhi; Baotou; Wuhu; Nanping; Fuzhou; Laiwu; Changsha, Xiangtan, Yueyang, Huaihua, Xiangxi Tujia and Miao Autonomous Prefecture; Zhanjiang, Heyuan, Yangjiang; Yulin; Longnan | Shanxi (1), Neimenggu (1); Anhui (1), Fujian (1), Jiangxi (1), Shandong (1); Hunan (5), Guangdong (3), Guangxi (1); Gansu (1) | North (2); East (4); South Central (9); Northwest (1) |
| 4 | 14 | Tangshan, Handan, Xingtai, Baoding, Hengshui; Harbin, Qiqihar, Hegang, Shuangyashan, Daqing, Yichun, Mudanjiang, Heihe, Suihua | Hebei (5); Heilongjiang (9) | North (5); Northeast (9) |
| 5 | 11 | Xinganmeng; Fushun, Dandong, Panjin, Huludao; Changchun, Jilin, Liaoyuan, White; Haikou, Directly under the provincial level administrative divisions in Hainan | Neimenggu (1); Liaoning (4), Jilin (4); Hainan (2) | North (1); Northeast (8); South Central (2) |
| 6 | 10 | Jinan; Urumqi, Karamay, Turpan area, Hami region, Changji Hui Autonomous Prefecture, Boertala Mongolian Autonomous Prefecture, Bayinguoleng Mongol Autonomous Prefecture, Tacheng, Altay region | Shandong (1); Xinjiang (9) | East (1); Northwest (9) |
| 7 | 9 | Bayannao'er; Shantou, Jiangmen, Huizhou; Chongqing area; Neijiang, Nanchong, Guang'an, Ziyang | Neimenggu (1); Guangdong (3); Chongqing (1), Sichuan (4) | North (1); South Central (3); Southwest (5) |
| 8 | 6 | Alxa League; Zhuhai, Qingyuan; Miao and Dong Autonomous Prefecture; Wuwei, Pingliang | Neimenggu (1); Guangdong (2); Guizhou (1); Gansu (2) | North (1); South Central (2); Southwest (1); Northwest (2) |
| 9 | 6 | Hangzhou, Ningbo, Shaoxing, Taizhou; Qingdao; Anyang | Zhejiang (4), Shandong (1); Henan (1) | East (5); South Central (1) |
| 10 | 4 | Bengbu, Huaibei, Chuzhou; Zhangjiajie | Anhui (3); Hunan (1) | East (3); South Central (1) |
| 11 | 4 | Ordos; Xiaogan; Panzhihua, Liangshan Yi Autonomous Prefecture | Neimenggu (1); Hubei (1); Sichuan (2) | North (1); South Central (1); Southwest (2) |
| 12 | 4 | Tongliao; Shenyang, Tieling; Songyuan | Neimenggu (1); Liaoning (2), Jilin (1) | North (1); Northeast (3) |
| 13 | 3 | Xuzhou, Yancheng; Shangqiu | Jiangshu (2); Henan (1) | East (2); South Central (1) |
| 14 | 3 | Huaian, Yangzhou, Suqian | Jiangshu (3) | East (3) |
| 15 | 3 | Jiayuguan, Dingxi; Haidong | Gansu (2), Qinghai (1) | Northwest (3) |
| 16 | 3 | Dalian; Putian; Nanchang | Liaoning (1); Fujian (1), Jiangxi (1) | Northeast (1); East (2) |
| 17 | 3 | Liuzhou, Baise; Jinchang | Guangxi (2); Gansu (1) | South Central (2); Northwest (1) |
| 18 | 3 | Loudi; Guangzhou; Dazhou | Hunan (1), Guangdong (1); Sichuan (1) | South Central (2); Southwest (1) |
| 19 | 3 | Shijiazhuang, Langfang; Jiamusi | Hebei (2); Heilongjiang (1) | North (2); Northeast (1) |
| 20 | 3 | Aksu Prefecture, Kashi Prefecture, Ili Kazak Autonomous Prefecture | Xinjiang (3) | Northwest (3) |
| 21 | 2 | Hengyang, Yongzhou | Hunan (2) | South Central (2) |
| 22 | 2 | Wuxi; Puyang | Jiangshu (1); Henan (1) | East (1); South Central (1) |
| 23 | 2 | Fuxin; Jingzhou | Liaoning (1); Hubei (1) | Northeast (1); South Central (1) |
| 24 | 2 | Tai'an; Shizuishan | Shandong (1); Ningxia (1) | East (1); Northwest (1) |
| 25 | 2 | Yingkou; Siping | Liaoning (1), Jilin (1) | Northeast (2) |
| 26 | 2 | Southwest Guizhou Buyi and Miao Autonomous Prefecture; Qingyang | Guizhou (1); Gansu (1) | Southwest (1); Northwest (1) |
| 27 | 2 | Leshan, Meishan | Sichuan (2) | Southwest (2) |
