# Supplementary material for: Geospatial characteristics of measles transmission in China during 2005−2014
Source: PLoS Comput Biol. 2017 Apr 4;13(4):e1005474. doi: 10.1371/journal.pcbi.1005474 (PMC5395235; doi:10.1371/journal.pcbi.1005474)
Supplement: S3 Table — (DOCX) [file pcbi.1005474.s003.docx]

**Table S3.** City clusters with synchronous epidemic cycles. Cities with a Pearson correlation coefficient *r*≥0.90 were identified as within the same cluster. The 1^st^ column shows the cluster id number, the 2^nd^ shows the total number of cities included in each cluster, the 3^rd^ lists the cities in each cluster, the 4^th^ lists the province(s) in each cluster, and the 5^th^ lists the region(s) in each cluster. The numbers in the parentheses in the 4^th^ and 5^th^ columns indicate the numbers of cities located in each province or region.

| **No.** | **# cities** | **Cities** | **Province(s)** | **Region(s)** |
| --- | --- | --- | --- | --- |
| 1 | 8 | Nantong; Jiaxing, Huzhou, Jinhua, Quzhou, Lishui; Tongchuan, Yan'an | Jiangshu (1), Zhejiang (5); Shaanxi (2) | East (6); Northwest (2) |
| 2 | 5 | Handan, Xingtai, Baoding; Mudanjiang, Suihua | Hebei (3); Heilongjiang (2) | North (3); Northeast (2) |
| 3 | 4 | Karamay, Turpan area, Hami region, Altay region | Xinjiang (4) | Northwest (4) |
| 4 | 4 | Neijiang, Nanchong, Guang'an, Ziyang | Sichuan (4) | Southwest (4) |
| 5 | 4 | Changji Hui Autonomous Prefecture, Boertala Mongolian Autonomous Prefecture, Bayinguoleng Mongol Autonomous Prefecture, Tacheng | Xinjiang (4) | Northwest (4) |
| 6 | 3 | Changzhi; Baotou; Fuzhou | Shanxi (1), Neimenggu (1); Jiangxi (1) | North (2); East (1) |
| 7 | 3 | Chifeng; Guyuan, Zhongwei | Neimenggu (1); Ningxia (2) | North (1); Northwest (2) |
| 8 | 3 | Aksu Prefecture, Kashi Prefecture, Ili Kazak Autonomous Prefecture | Xinjiang (3) | Northwest (3) |
| 9 | 3 | Hegang, Daqing, Heihe | Heilongjiang (3) | Northeast (3) |
| 10 | 2 | Anqing; Zhangye | Anhui (1); Gansu (1) | East (1); Northwest (1) |
| 11 | 2 | Miao and Dong Autonomous Prefecture; Wuwei | Guizhou (1); Gansu (1) | Southwest (1); Northwest (1) |
| 12 | 2 | Changzhou, Suzhou | Jiangshu (2) | East (2) |
| 13 | 2 | Shaoxing, Taizhou | Zhejiang (2) | East (2) |
| 14 | 2 | Shannan Prefecture; Golog Tibetan Autonomous Prefecture | Tibet (1); Qinghai (1) | Southwest (1); Northwest (1) |
