# Supplementary figures and images for: Geospatial characteristics of measles transmission in China during 2005−2014
Source: PLoS Comput Biol. 2017 Apr 4;13(4):e1005474. doi: 10.1371/journal.pcbi.1005474 (PMC5395235; doi:10.1371/journal.pcbi.1005474)

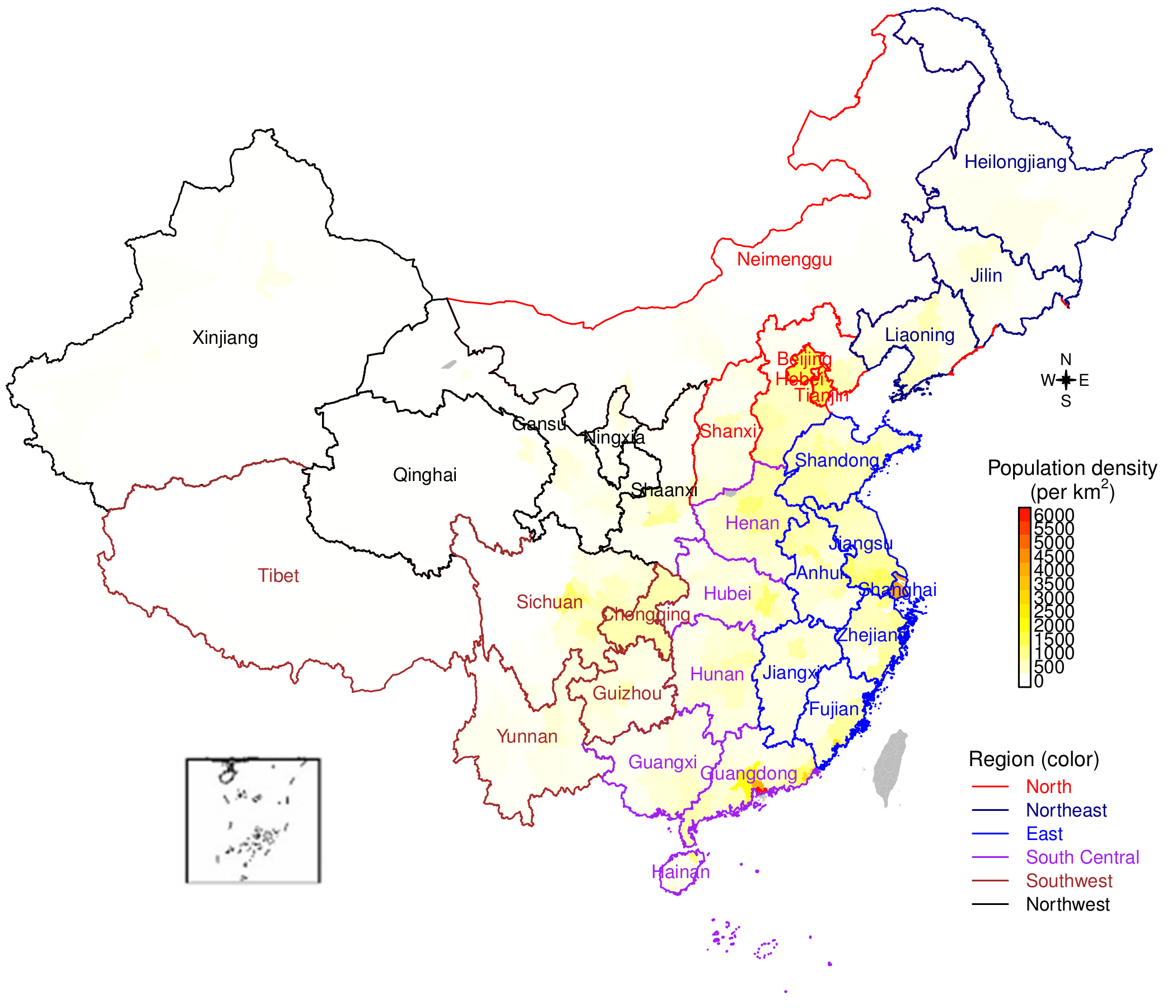

Supplement: S1 Fig — There are six geographical regions in China; provinces in the same region are labeled and outlined in the same color: North in red, Northeast in navy blue, East in blue, South Central in purple, Southwest in brown, and Northwest in black. Prefecture city level population density is shown by color as indicated in the legend. (TIF) [file pcbi.1005474.s004.tif]

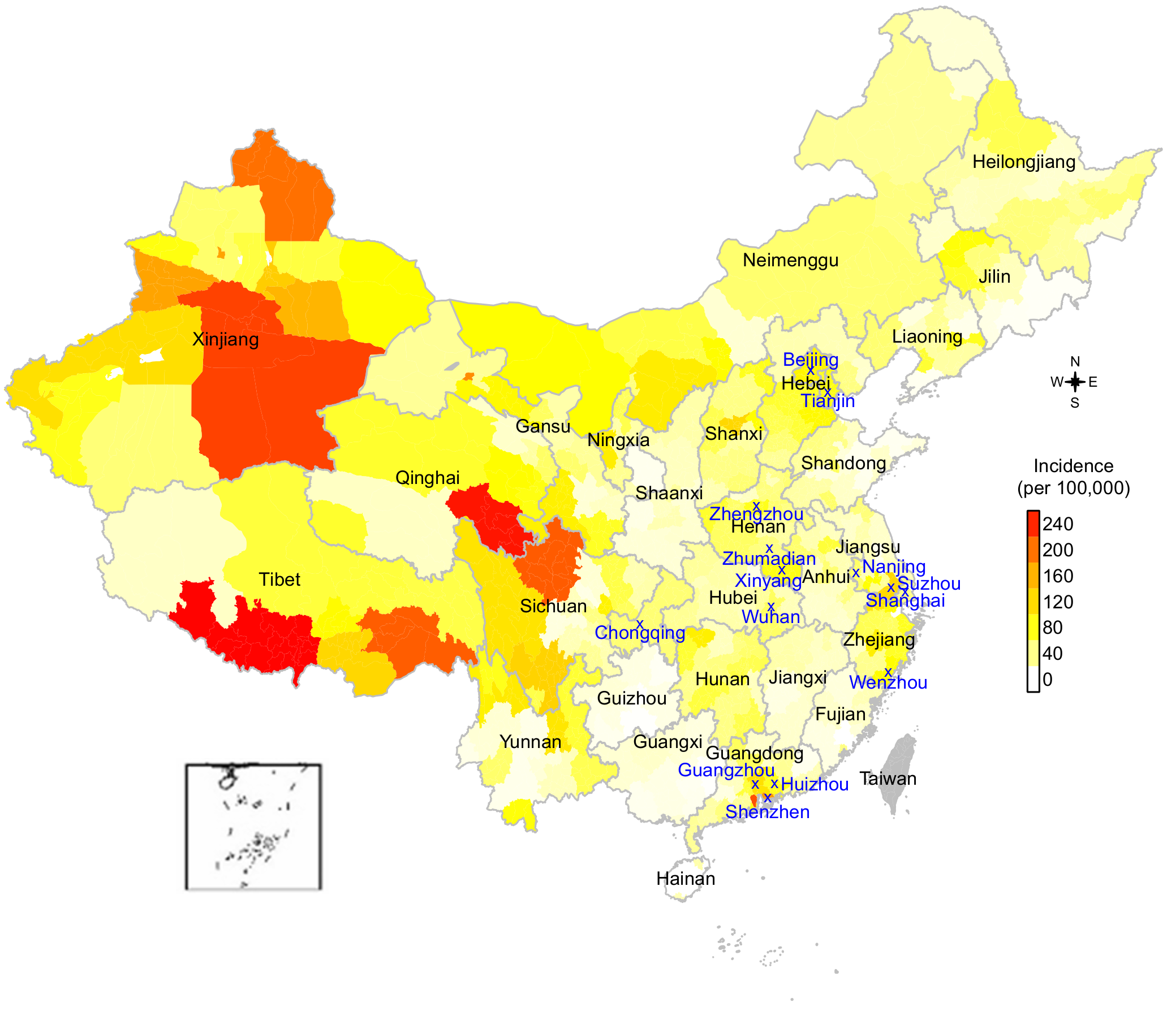

Supplement: S2 Fig — Cities labeled in blue and shown by an ‘x’ experienced endemic transmission during this phase. (TIF) [file pcbi.1005474.s005.tif]

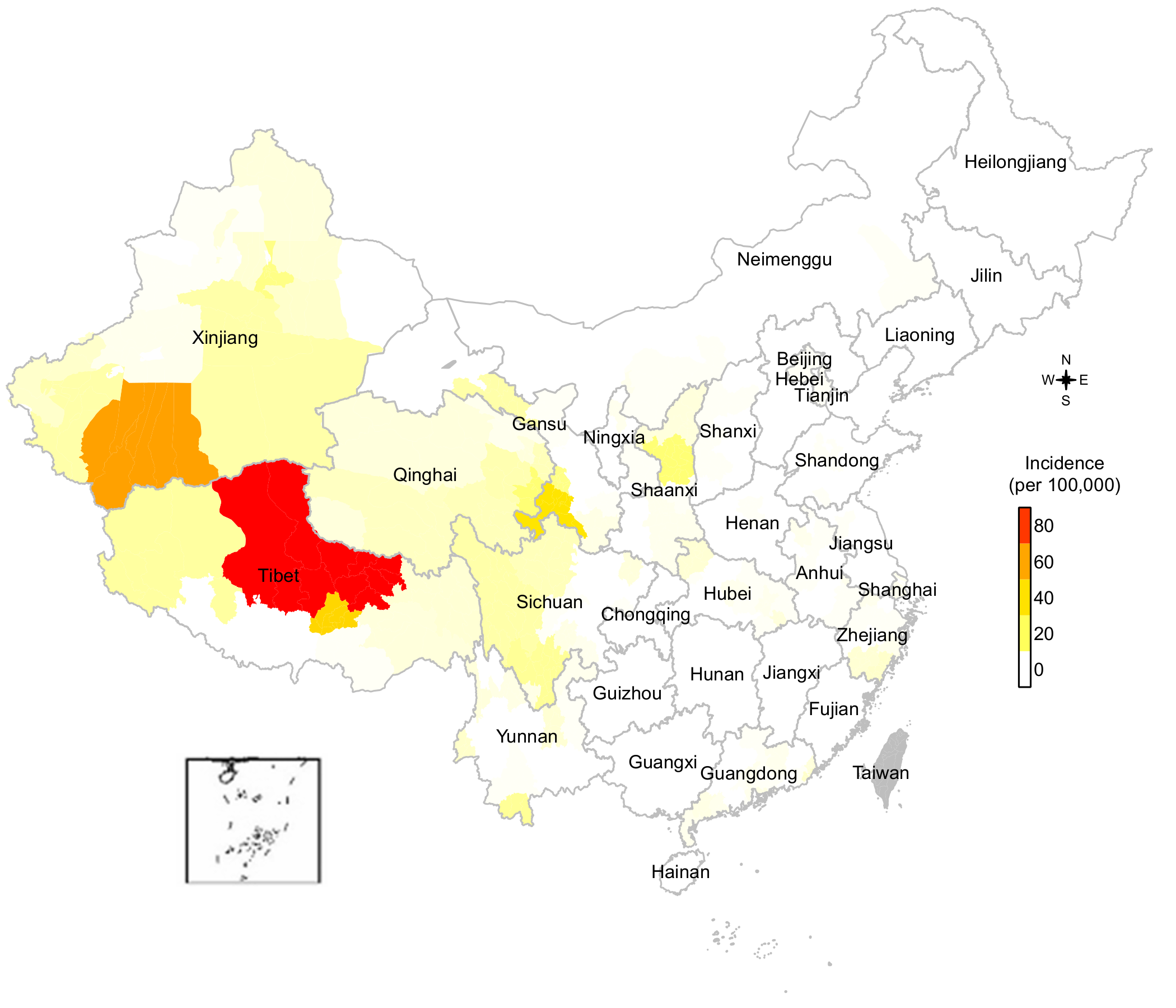

Supplement: S3 Fig — None of the 344 cities experienced endemic transmission during this phase. (TIF) [file pcbi.1005474.s006.tif]

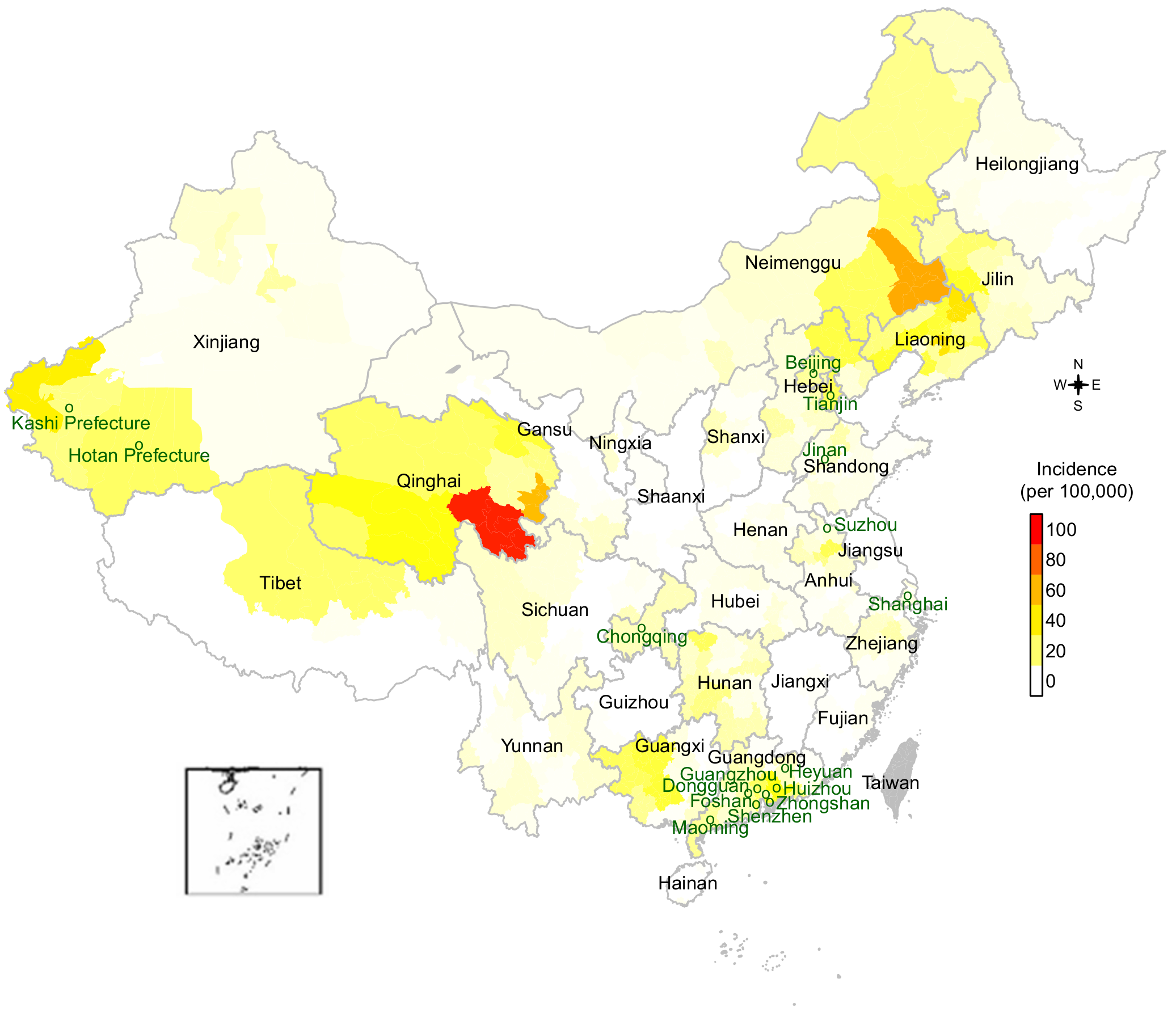

Supplement: S4 Fig — Cities labeled in green and shown by an ‘o’ experienced endemic transmission during this phase. (TIF) [file pcbi.1005474.s007.tif]

(A) Recursive Merging

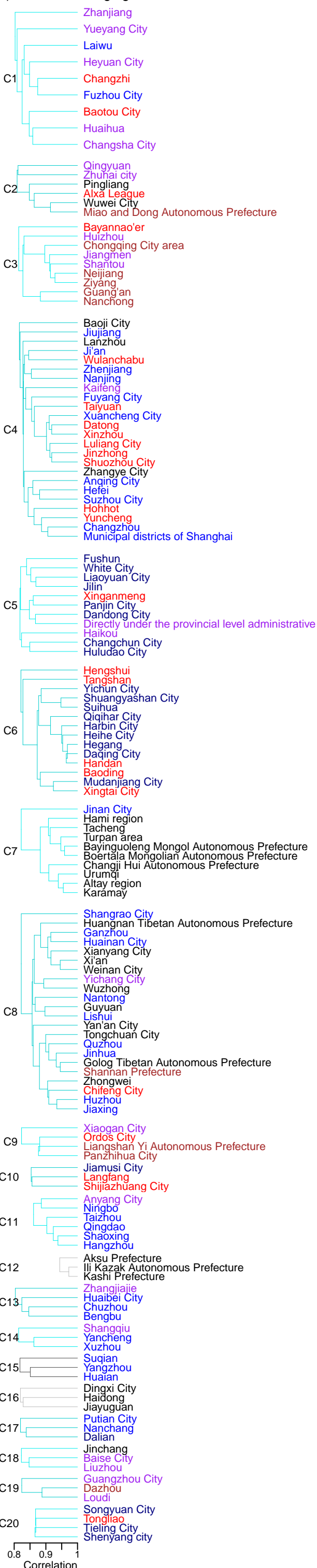

(B) hclust

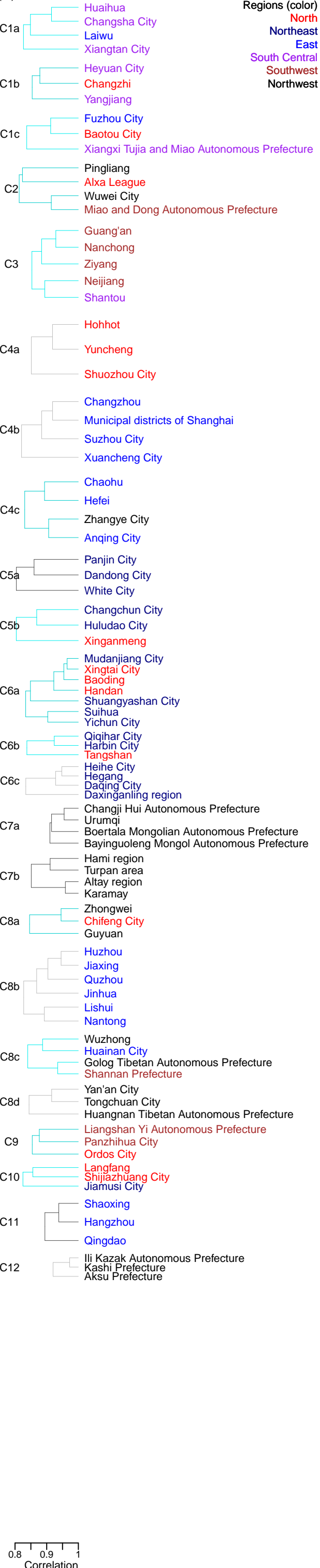

Supplement: S5 Fig — Only those with three or more cities are included here; a complete list of clusters is shown in S2 Table. (PDF) [file pcbi.1005474.s008.pdf]

(A) Recursive Merging

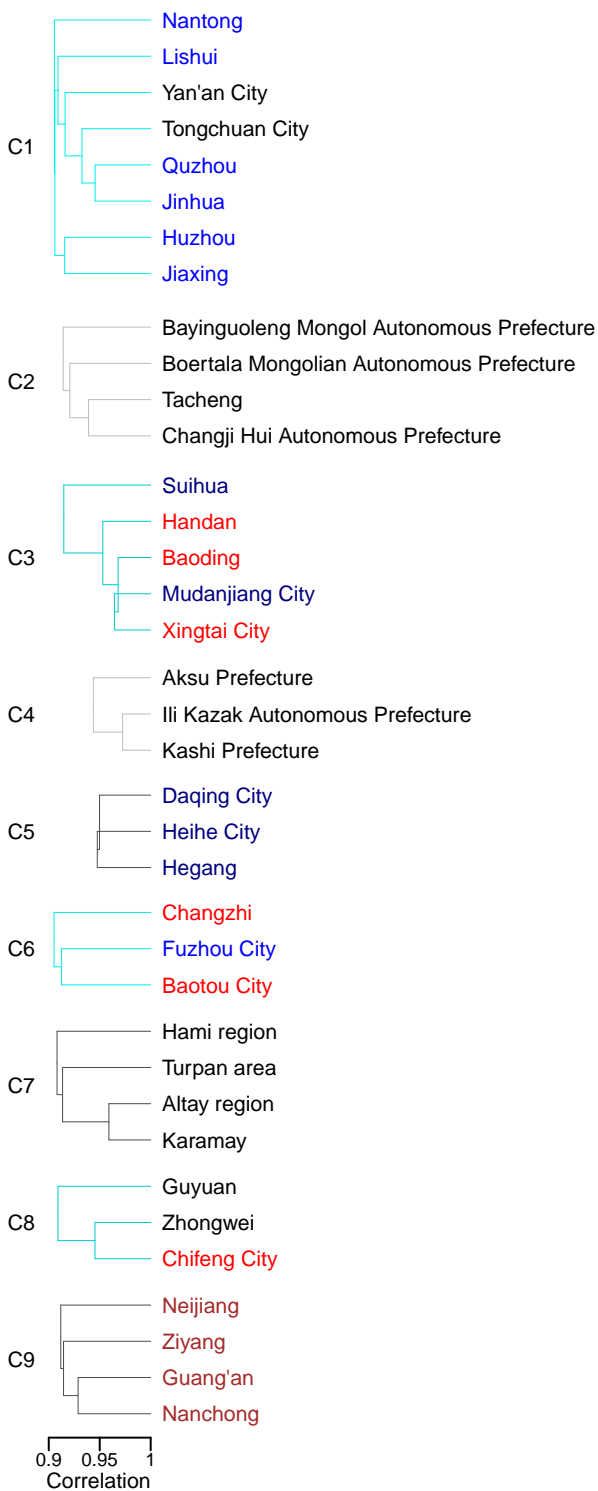

(B) hclust

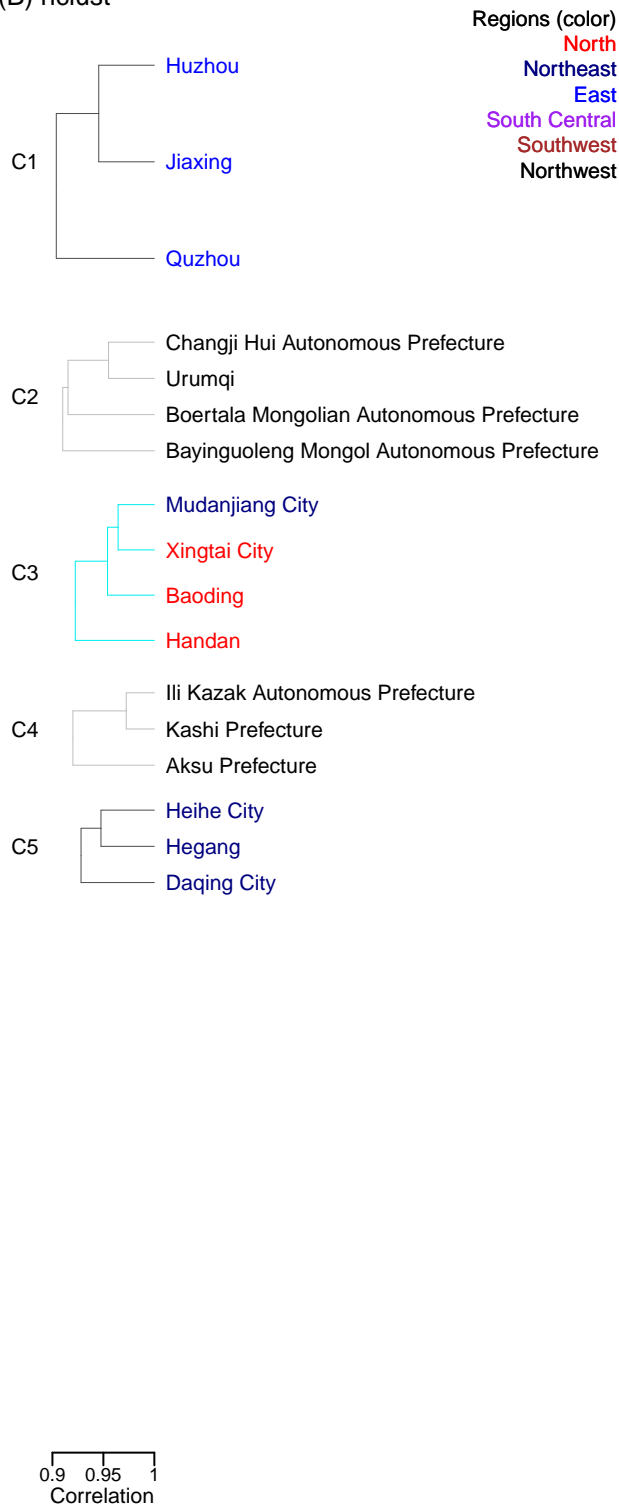

Supplement: S6 Fig — Only those with three or more cities are included here; a complete list of clusters is shown in S3 Table. (PDF) [file pcbi.1005474.s009.pdf]
